# Predicting Protein-Protein Interaction by the Mirrortree Method: Possibilities and Limitations

**DOI:** 10.1371/journal.pone.0081100

**Published:** 2013-12-13

**Authors:** Hua Zhou, Eric Jakobsson

**Affiliations:** 1 Department of Biochemistry, University of Illinois, Urbana-Champaign, Illinois, United States of America; 2 Beckman Institute, National Center for Supercomputing Applications, Program in Biophysics and Computational Biology, Department of Molecular and Integrative Physiology, University of Illinois, Urbana-Champaign, Illinois, United States of America; National Institutes of Health, United States of America

## Abstract

Molecular co-evolution analysis as a sequence-only based method has been used to predict protein-protein interactions. In co-evolution analysis, Pearson's correlation within the mirrortree method is a well-known way of quantifying the correlation between protein pairs. Here we studied the mirrortree method on both known interacting protein pairs and sets of presumed non-interacting protein pairs, to evaluate the utility of this correlation analysis method for predicting protein-protein interactions within eukaryotes. We varied metrics for computing evolutionary distance and evolutionary span of the species analyzed. We found the differences between co-evolutionary correlation scores of the interacting and non-interacting proteins, normalized for evolutionary span, to be significantly predictive for proteins conserved over a wide range of eukaryotic clades (from mammals to fungi). On the other hand, for narrower ranges of evolutionary span, the predictive power was much weaker.

## Introduction

Proteins seldom act alone; rather, they tend to carry out their activities via interactions or networks. The detection of protein interactions can help to better understand the molecular machinery of the cell and expose biological processes and pathways that have not been characterized so far. Thus, to understand the mechanism of proteins; it's important to study their partners as well. In recognition of this importance, there are several public protein-protein interaction databases available online, for example DOMINE [1], Biogrid [2], String [3], MIMI [4], DIP [5], etc. However the databases are far from complete, necessitating the prediction of interactions not yet in the databases.

Traditionally, protein-protein interactions have been studied via wet-lab experimental methods, such as yeast two-hybrid [6] and mass spectrometry [7]–[8]. These are high-throughput technologies but also expensive and time-consuming. On the other hand, techniques such as affinity chromatography [9] and co-immunoprecipitation [8] are low-throughput methods. The availability of comprehensive protein sequences for many organisms makes it possible to attempt an in silico system-level study of protein interactions in the hope of deriving an efficient and low-cost high-throughput method to augment experimental methods.

Methods for computational prediction of protein-protein interactions can be mainly classified to two different approaches: studies that use structural information [10] and co-evolution analysis based entirely on sequence [11]–[12]. Co-evolution analysis can be applied to whole protein level or domain level to infer possible interactions. Natarajan et al. [13] applied coevolution analysis to the K_v_1.2-β_2_ complex using 9 mer sliding windows, to infer the composition of a control network interacting with the complex via domain-domain interactions.

Co-evolutionary analysis for whole proteins can be based on either codon usage or amino acid sequences. Fraser et al. [14] used the Codon Adaptation Index (CAI) based on codon usage to infer protein expression level and further used protein expression level as the signal for co-evolutionary study, but CAI is so far not readily applicable to multicellular organisms. The underlying logic of methods based on amino acid sequences is that substitution of an amino acid residue in one protein will select for the coordinated mutation of an amino acid in a second protein with which the first protein interacts. The nature of the interaction may be direct, as in participation in a multi-protein complex, or indirect, as in being in the same network or pathway. The mirrortree method utilizing this logic has been developed to predict protein interaction partners and functional relationships [15]–[19] in a wide range of organisms. In this study we examine the efficacy of mirrortree as applied to eukaryotes, as a function of different parameters of calculation.

The mirrortree method consists of the following steps: 1). find orthologs of the two proteins in multiple species, 2). align the ortholog sequences from the common species to get a multiple sequence alignment (MSA), 3). Create an evolutionary distance matrix either directly from the pairwise evolutionary distances between the aligned protein pairs or from a phylogenetic tree constructed from the MSA, and 4). Construct a linear correlation coefficient (Pearson's correlation) to determine the co-evolution of protein pairs and further predict possible interactions.

Recent studies using mirrortree method to infer protein-protein interactions include the following: Kann et al. [20] and Hakes et al. [21] examined the different degrees of correlation in binding regions and the whole protein sequences. However the two studies reached different conclusions. Hakes et al. found that the degree of correlation was no higher in the binding interfaces than in the whole sequence of the protein, while Kann et al. found that degree of correlation was significantly higher in the binding interfaces. Since the methodology was essentially the same, we infer that the different results pertain to the selection of datasets. In the Hakes et al. study, different ortholog pairs were from different species sets, whereas in the Kann et al. study, all the ortholog pairs were from the same set of species. Juan et al. [19] extended the mirrortree method by considering genome-wide context of interactions rather than interacting pairs in isolation. Herman et al. [22], working entirely within bacteria and archaea, studied the effect of different choices of organism set on the performance of mirrortree and related methods. Clark et al. [23] suggested that better prediction performance could be gained by choosing submatrices rather than complete matrices of all orthologous sequences (MMM method).

The effect of species genome choice on the efficacy of mirrortree-like methods has been evaluated for bacterial and archaeal genomes [22], [24]. In this paper we extend the assessment to eukaryotic genomes, specifically considering the effects of evolutionary distance spanned by the genomes on co-evolution analysis.

## Materials and Methods

The key relationship defining the correlation between two sets of protein orthologs is the Pearson's correlation coefficient, given in equation (1).
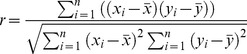
(1)Here X and Y designate sets of orthologous proteins whose interaction propensity we wish to predict. 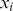 and 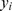 are the pairwise distances between orthologs. For example, if we have n orthologous proteins, the number of pairwise distances is n(n-1)/2. The sets X and Y come from the same species. 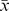 and 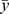 are the mean values of all the 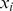 and 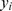 respectively. “
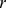
” is the extent to which evolutionary variations in 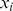 and 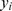 are correlated with each other.

### Datasets

We used the Biogrid database because it contains large sets of functional related or directly interacting protein pairs categorized into different species. In particular we used datasets from human and Saccharomyces cerevisiae (Baker's Yeast) as a standard to define interacting protein pairs. To identify orthologs of the proteins, we use the results of the OMA project [25] since it has a relatively comprehensive orthology dataset with 6.2 million proteins from 1,320 species. As a control, non-interacting protein pair sets were generated by random shuffling of the interacting protein pairs. The choice of human and S. cerevisiae was to provide as wide as possible an evolutionary span among the eukaryotes, to make sure the analyzed sets have the largest possible variation for comparing the difference of correlation between interacting and non-interacting protein pairs. In addition, S. cerevisiae and human are intensively studied species with a large number of known protein interacting pairs, with 218,492 and 131,624 non-redundant interacting protein pairs respectively listed in Biogrid. At this writing there are a total of 28,659 human proteins and 6,328 S. cerevisiae proteins listed with ortholog groups in OMA. Between human and S. cerevisiae, there are 2,012 common proteins in OMA.

Common interacting protein pairs are retrieved from interacting datasets of human and S. cerevisiae species. A total of 1,062 common interacting protein-protein pairs were found in the Biogrid data base from human and S. cerevisiae. Of these, 311 protein pairs were found to have corresponding ortholog groups in OMA browser. Adding the criterion that each group to be compared should have 15 or more common species in the common ortholog sets reduced the membership of the set for analysis from 311 to 259.

We created a second set of putative interacting pairs by including all human Biogrid interaction pairs whose members have S. cerevisiae orthologs plus all S. cerevisiae interacting pairs whose members have human orthologs. The difference between the first set and the second set is that in the first set both the human and the S. cerervisiae pairs are confirmed experimentally to interact, whereas in the second set the interaction needed to be confirmed experimentally in only one of the two. Both datasets were constrained by the requirement that every protein needed to have 15 or more common species in their OMA ortholog sets. The total number of pairs fulfilling the requirements for the second set was 5,616. Finally a set of 5,616 different non-interacting pairs were created by sampling the second set with replacement [26], coupled by filtering to discard accidental coupling of interacting pairs and duplications. We call the first set of interacting pairs plus the constructed set of non-interacting pairs Dataset 1. We call the second set of interacting pairs plus the constructed set of non-interacting pairs Dataset 2.

We also created a third dataset of human and mouse common interacting protein pairs, using a procedure exactly analogous to the procedure for creating Dataset 1 (human and yeast set). This procedure gives us a total of 1,375 interacting protein pairs. We created a corresponding non-interacting set of 5,630 pairs by sampling with replacement and filtering in the same fashion as we did for Dataset 2. This set of 1,375 putative interacting pairs plus the 5,630-member set of non-interacting pairs we call Dataset 3.

For each protein from the interacting or non-interacting protein pairs, we retrieved its 1 to 1 ortholog groups containing different species from OMA database. For each protein pair we extracted the common species to the 2 ortholog groups and used the 2 ortholog groups with common species set for evolutionary distance calculations.

### Evolutionary distance calculations

All the protein pairs datasets were aligned using MUSCLE (Multiple sequence comparison by log-expectation) [27].

The pair-wise distances for sequences from different species for any protein were calculated using the protdist package [28]. We experimented with four different distance measures:

1. Jones Taylor Thornton matrix [29].
2. Dayhoff Pam Matrix which uses Dayhoff's PAM 001 matrix [30].
3. Kimura model, in which distance is defined as:

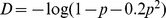
, here 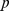 defines the fraction of difference for 2 sequences.
4. Categories Model [31]–[32], in which amino acids are lumped into the following categories: Group 1, sulfhydryl: cysteine; Group 2, small/neutral: serine, threonine, alanine, proline and glycine; Group 3, acidic: aspartate, glutamate, asparagine and glutamine; Group 4, basic: histidine, arginine and lysine; Group 5, hydrophobic: valine, leucine, isoleucine and methionine; and Group 6, aromatic: phenylalanine, tyrosine, and tryptophan. There is no penalty for a substitution within a group and 0.457 for a substitution of a member of one group for a member of another group.

We also explored the use of other measures for evolutionary distance, including unweighted direct sum (0 or 1 for same or different) of position-specific substitutions, and different PAM or BLOSUM matrices with various ways of treating gap penalties. All results were essentially independent of the type of evolutionary distance employed, so in results we report using only one distance measure, the Jones Taylor Thornton matrix.

### Other Factors

For further study of species coverage effects on correlation analysis, we started with the human and mouse interacting protein pairs' datasets (Dataset 3) with 1,375 interacting and 5,630 non-interacting protein pairs. Then we divided the data into three categories: 1) present only in chordates (834 pairs); 2) present in other metazoan as well as in chordates, but not in plants or fungi (349 pairs); 3) present in all the eukaryotic kingdoms (192 pairs). The results of analysis of this dataset will be shown in the Results and Discussion section.

## Results and Discussion

### Assessment of co-evolution

**Figure 1:**
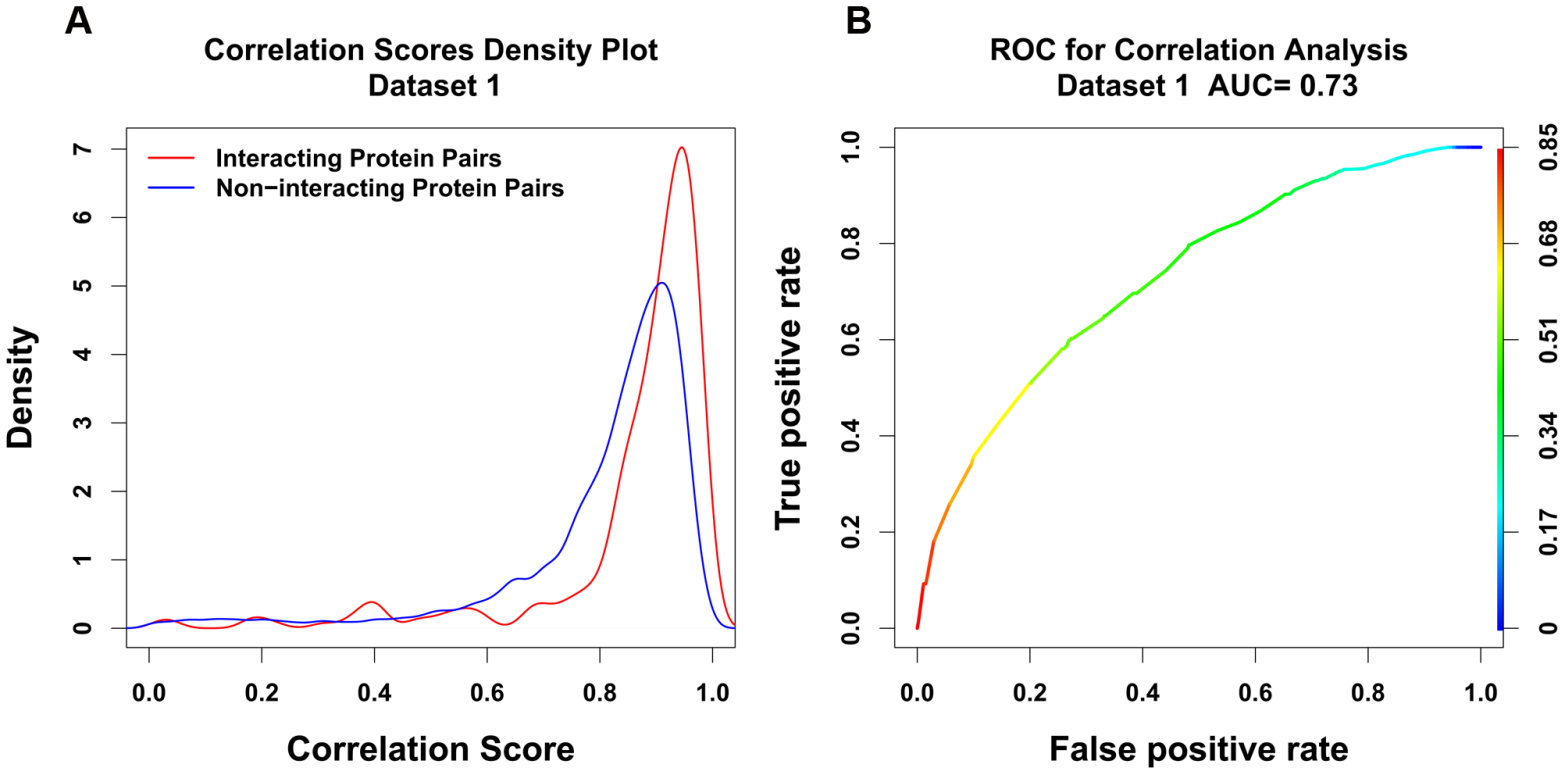
Density plot for correlation scores using Jones-Taylor-Thornton matrix for common interacting and non-interacting protein pairs from Dataset 1 (A) and the corresponding ROC plot (B).

To illustrate the differences of correlations of interacting versus non-interacting protein pairs, the correlation scores for interacting and non-interacting protein-protein pairs were plotted against each other. Figure 1A shows smoothed histograms (density plots of incidence) of the correlation scores from the Biogrid protein pairs common to human and S. cerevisiae. The correlation scores peaked at 0.95 and 0.91 for interacting and non-interacting protein-protein pairs respectively. A separation of non-interacting from interacting protein-protein pairs is evident. To evaluate the prediction power of our correlation analysis, the receiver operating characteristic (ROC) curve [33] was plotted and showed a clear view of prediction power (Figure 1B), an AUC of 0.73.

An AUC score of 0.5 would indicate no predictive power, while a score of 1.0 would indicate perfect predictive power. Therefore a score of .73 indicates significant, but not perfect, predictive power. The right side edge shows the cut-off correlation score for prediction, scaled by color. We can read the corresponding true positive and false positive rates from the curve by matching the color in the curve to the right side correlation score color scale.

**Figure 2:**
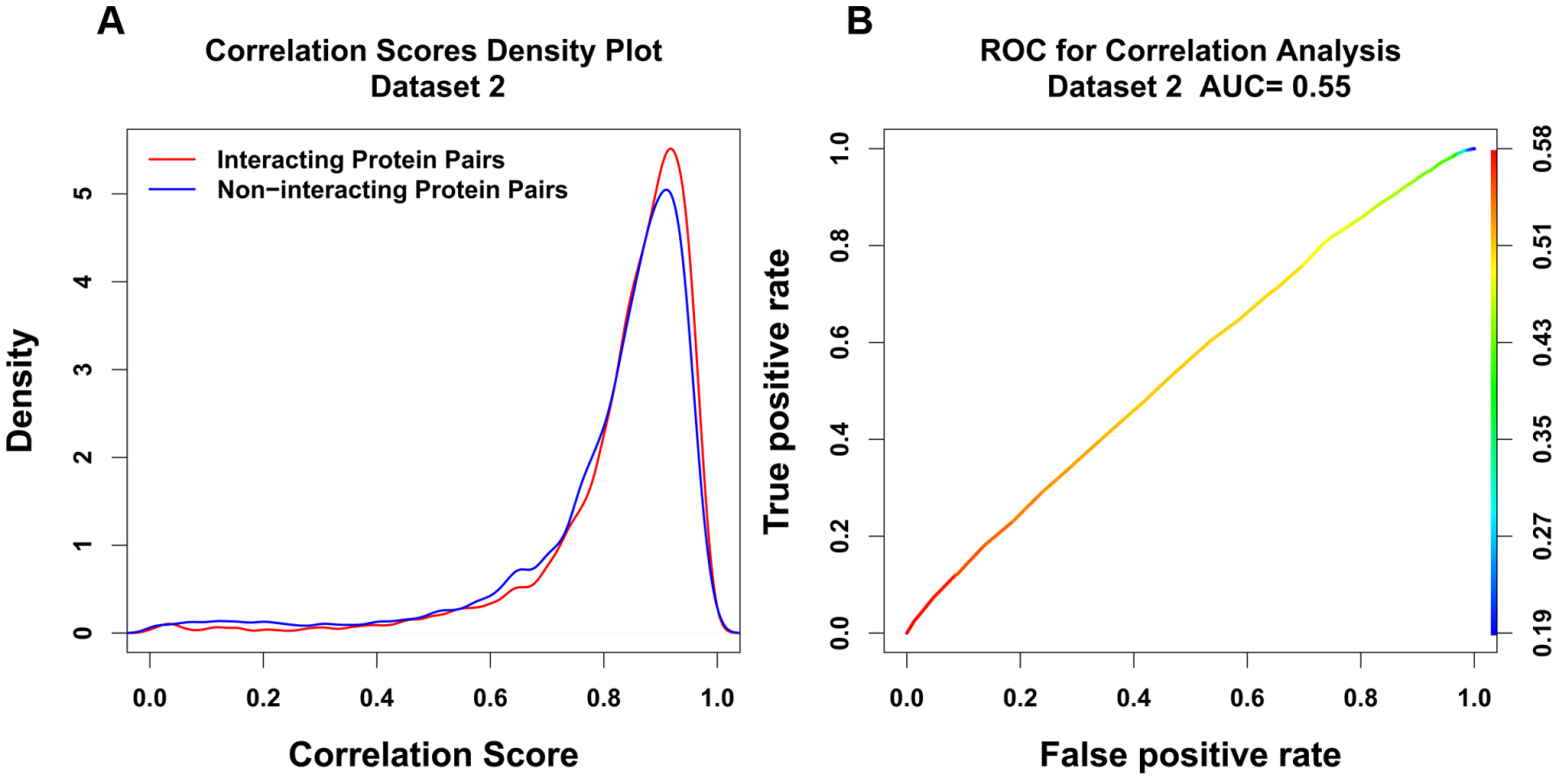
Density plot for correlation scores using Jones-Taylor-Thornton matrix for common interacting and non-interacting protein pairs from Dataset 2 (A) and the corresponding ROC plot (B).

We then studied the interacting protein pairs from human or S. cerevisiae (Dataset 2), the only difference of Dataset 1 from Dataset 2 was that the interaction in Dataset 2 was not necessary conserved in both human and S. cerevisiae. Density plot and ROC plot were plotted here too shown in Figure 2A and 2B respectively. Looking into the density plot (Figure 2A), there was no clear separation of interacting from non-interacting protein pairs. Both of them peaked at correlation score of around 0.90. ROC plot (Figure 2B) shows an AUC score of 0.55, which tells no significant separation either. Comparing Figure 1 and Figure 2, we concluded that independent evidence of conservation of interaction across species is an important determinant of the performance of co-evolutionary analysis, and should be considered when doing prediction.

**Figure 3:**
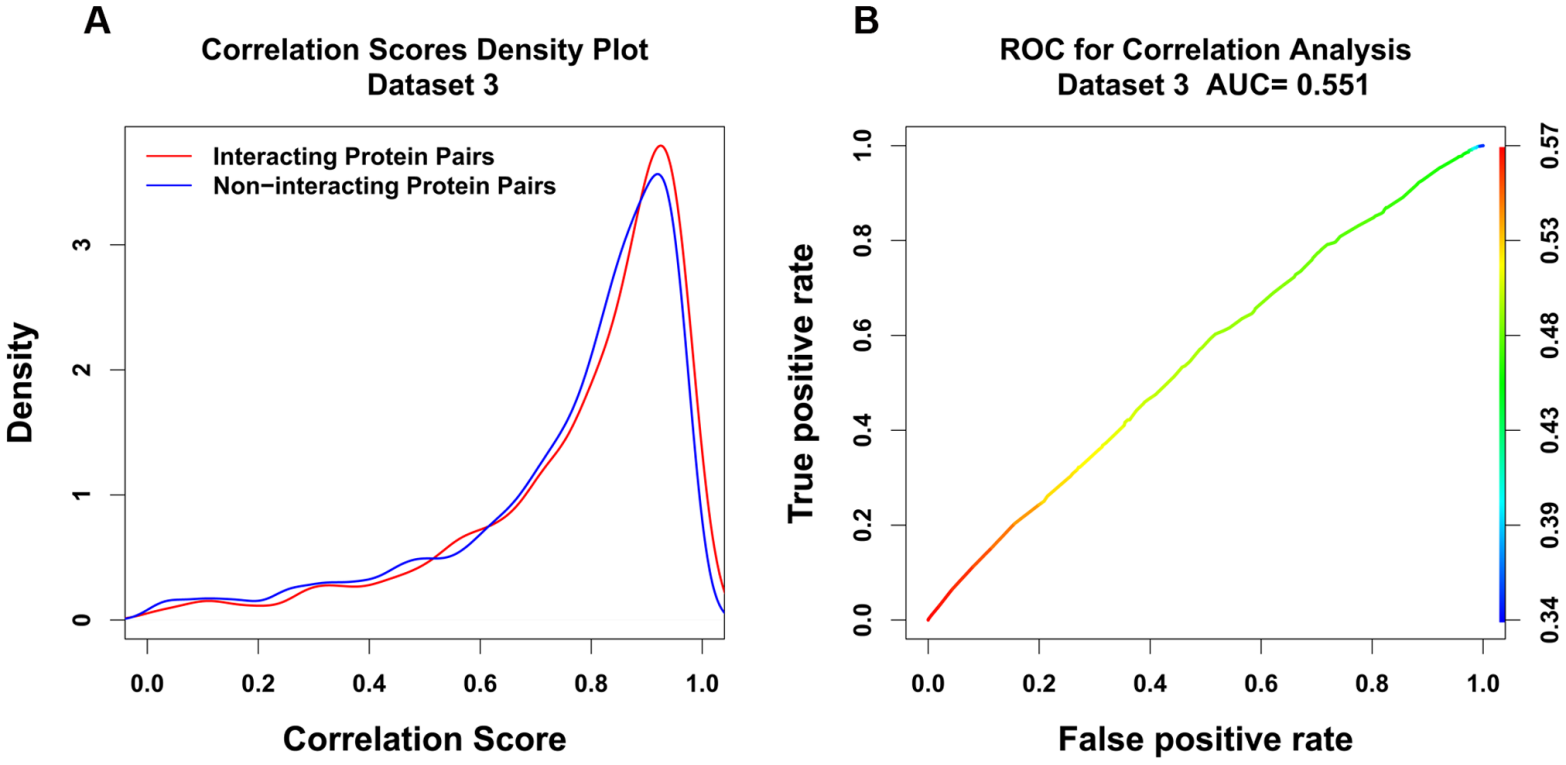
Density plot for correlation scores using Jones-Taylor-Thornton matrix for common interacting and non-interacting protein pairs from Dataset 3 (A) and the corresponding ROC plot (B).

We also studied the common interacting protein pairs between human and mouse (Dataset 3). The correlation density plot and ROC curve were plotted and shown in Figure 3. In the density plot (Figure 3A) the curves of interacting protein pairs' density (in red) and non-interacting protein pairs' density (in blue) were almost superimposed on each other. The ROC curve (Figure 3B) also gives a relatively low AUC score of 0.55. The differences between Dataset 3 and Dataset 1 are two-fold. One difference is that the evidence for conservation of interaction in Dataset 3 is between two closely related species (human and mouse) while in Dataset 1 the evidence for conservation of interaction is between two distantly related species (human and yeast). The second difference is that in Dataset 1 the ortholog sets all spanned the range between human and yeast, while in Dataset 3 the evolutionary span was variable from one ortholog set to the other. Some spanned all the way to yeast, while others were contained only in metazoan, others only in chordates, and others only in mammals. The relatively low level of discrimination between interacting and non-interacting pairs in Dataset 3 suggests that evolutionary span is an important factor in using and interpreting the mirrortree method.

For a single measure of the predictive power of the method, we elected to use the Matthews correlation coefficient (MCC). MCC is a more robust measure of effectiveness of binary classification methods than such measures as precision, recall, and F-measure because it takes into account in a balanced way of all four factors contributing to the effectiveness; true positives, false positives, true negatives and false negatives. A good review of methods for binary classification is given in Powers, 2011 [34]. The MCC is given by:
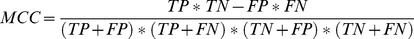
(2)Where

TP is the number of true positives

TN is the number of true negatives

FP is the number of false positives

FN is the number of false negatives.

**Figure 4:**
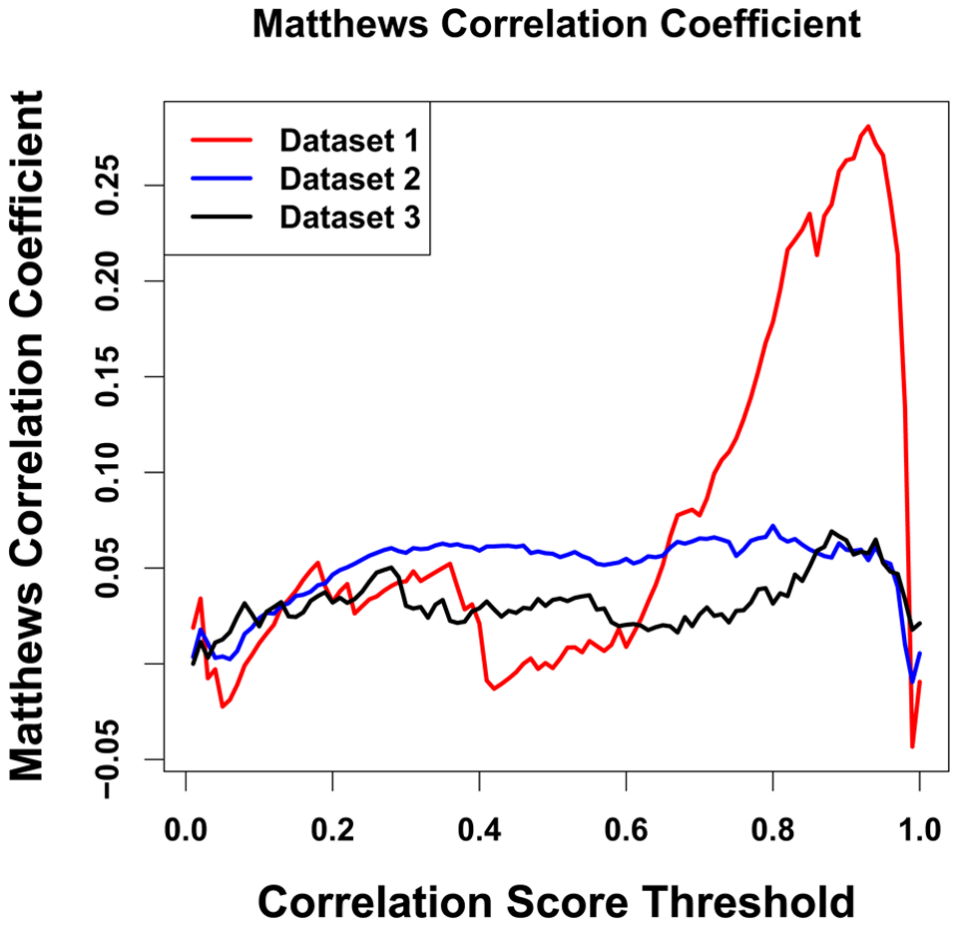
Matthews correlation coefficient (MCC) vs. choice of binary classification threshold for Datasets 1, 2, 3. It is seen that there is a much higher and more distinct peak for Dataset 1, supporting the inference derived from the relative AUC scores (Figures 1, 2, and 3) that the Dataset 1 provides the best differentiation between the interacting and non-interacting pairs.

In Figure 4, we plotted the Matthews correlation coefficient against its corresponding correlation score threshold for all 3 different sets. We can see the Human and Yeast set (Dataset 1) gives highest Matthews correlation coefficient and a distinct peak at a correlation score of approximately 0.9. A reasonable interpretation of the MCC is that a good choice for the threshold of the classification is at the peak of the MCC, while a good measure of the efficacy of the method is the height of the peak. In Dataset 2, on the other hand, there is no peak but rather a wide plateau with a relatively low height. Dataset 3 shows a peak, but a relatively low one, indicating a relatively weak binary classification efficacy. The MCC results are consistent with the results of the ROC curves (Figures 1–3) in suggesting that the mirrortree method has much better binary classification efficacy for Dataset 1 than for the other two.

**Figure 5:**
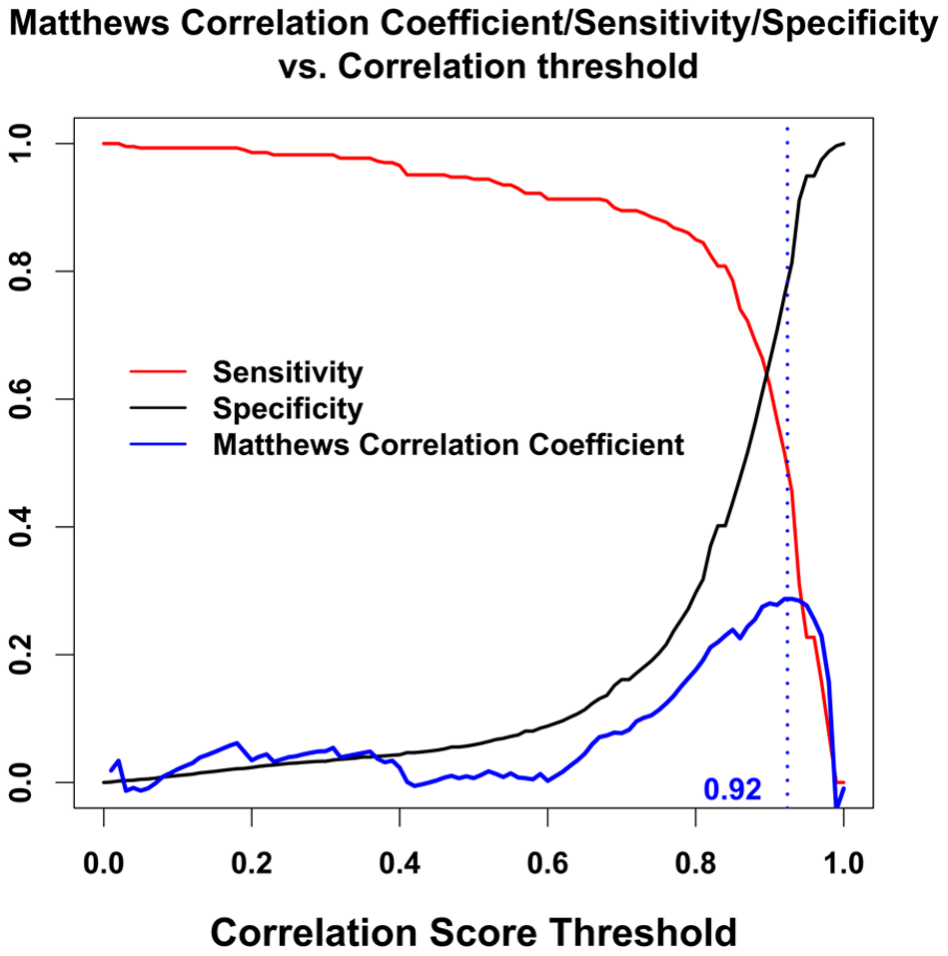
Plot of sensitivity, specificity, and MCC vs. threshold for binary classification using Dataset 1. It is seen that the peak of the MCC (dashed vertical line) occurs in this case where the specificity is somewhat larger than the sensitivity. A user may wish to use a threshold either larger or smaller than the position of the peak of the MCC, depending on whether specificity or sensitivity is more highly valued.

In Figure 5 we show the MCC vs. threshold on the same plot as sensitivity (TP/(TP+FN)) and specificity (TN/(TN+FP)) for Dataset 1. We see that the peak of the MCC occurs where the specificity is somewhat greater than the sensitivity. A user might move the classification threshold somewhat lower or higher depending on whether it is more important to retrieve all or practically all true positives, or whether it is rather more important to ensure that the positive results are not contaminated with false positives.

### Relationship of sequence degree of conservation and correlation score

**Figure 6:**
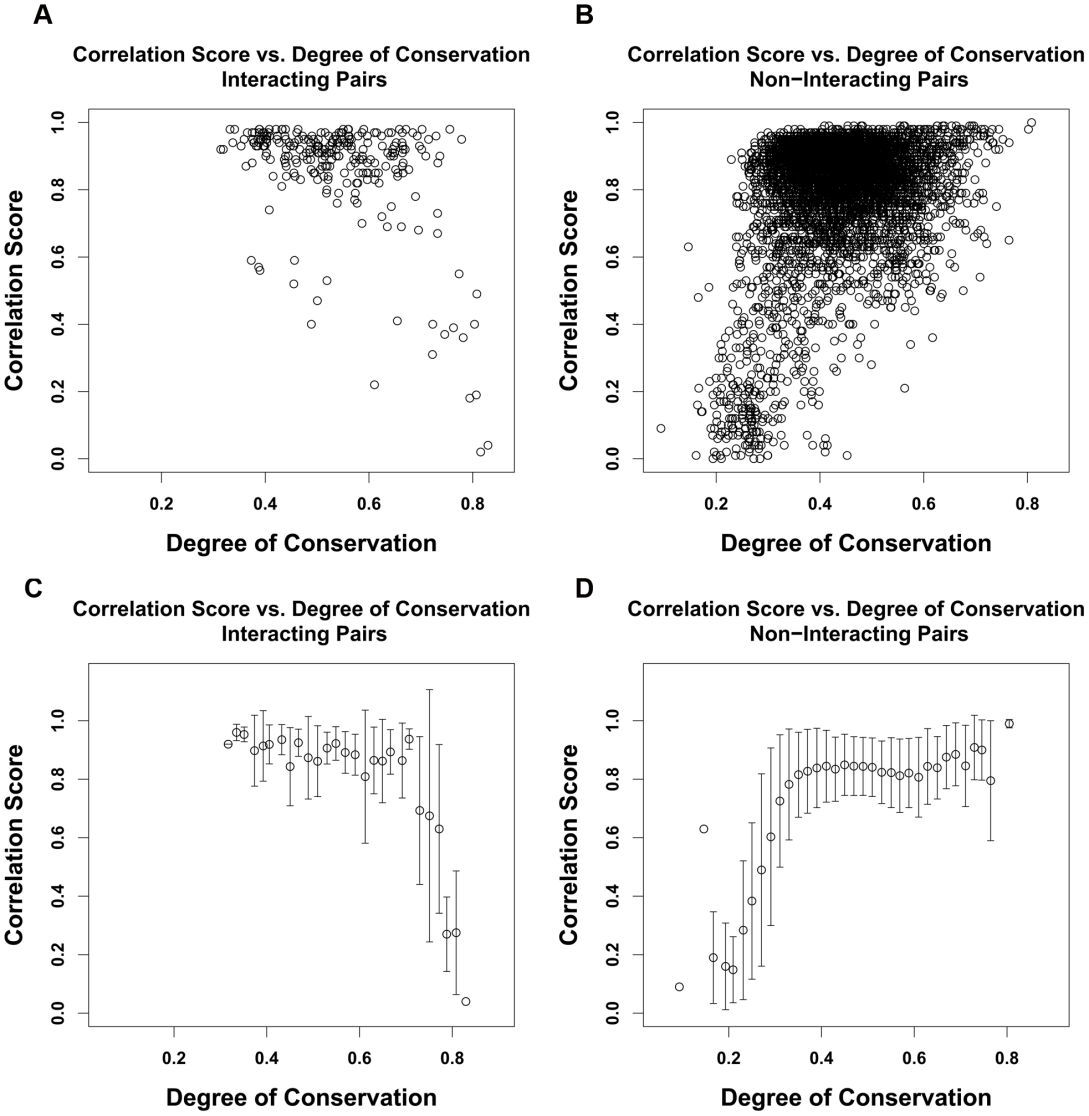
Protein sequences' within ortholog set degree of conservation (mean pairwise fraction identity for all orthologs in each set) vs. protein pairs correlation score for Dataset 1. A). Scatter plots of degree of conservation vs. protein pairs correlation score for interacting protein pairs. B). Scatter plots of degree of conservation vs. protein pairs correlation score for non-interacting protein pairs. C). Mean degree of conservation vs. protein pairs correlation score for interacting pairs with standard deviation as error bar. D). Mean degree of conservation vs. protein pairs correlation score for interacting pairs with standard deviation as error bar.

We note that for a set of completely random sequences the correlation scores will average zero. At the other extreme, for a set of identical sequences the Pearson's correlation score will be undefined. We accordingly wondered if, between these extremes, there would be any systematic dependence of correlation scores on total conservation of the pairs. To explore this, we started with the set of interacting and non-interacting protein pairs from human and S. cerevisiae species (Dataset 1). For each protein-pair's ortholog sets, we calculated the degree of conservation as the average identity for each pair of aligned sequences within each ortholog set, and then the average of the two means. The correlation score for each specific protein pair was calculated as stated in the method part. Figure 6 shows, for interacting pairs (6A) and for non-interacting pairs (6B) correlation scores vs. degree of conservation for all the ortholog pairs of Dataset 1. To see more clearly possible trends Figures 6C and 6D show mean correlation scores for sets binned in conservation score ranges of .02. We see that for the interacting set there are some pairs that have high conservation and low correlation score. These are responsible for the prominent bump at a correlation score of about 0.4 in the correlation distribution of the interacting pairs in Figure 1A. On the other hand for the non-interacting set, there are some pairs that have very low conservation and correlation.

### Evolutionary span

**Figure 7:**
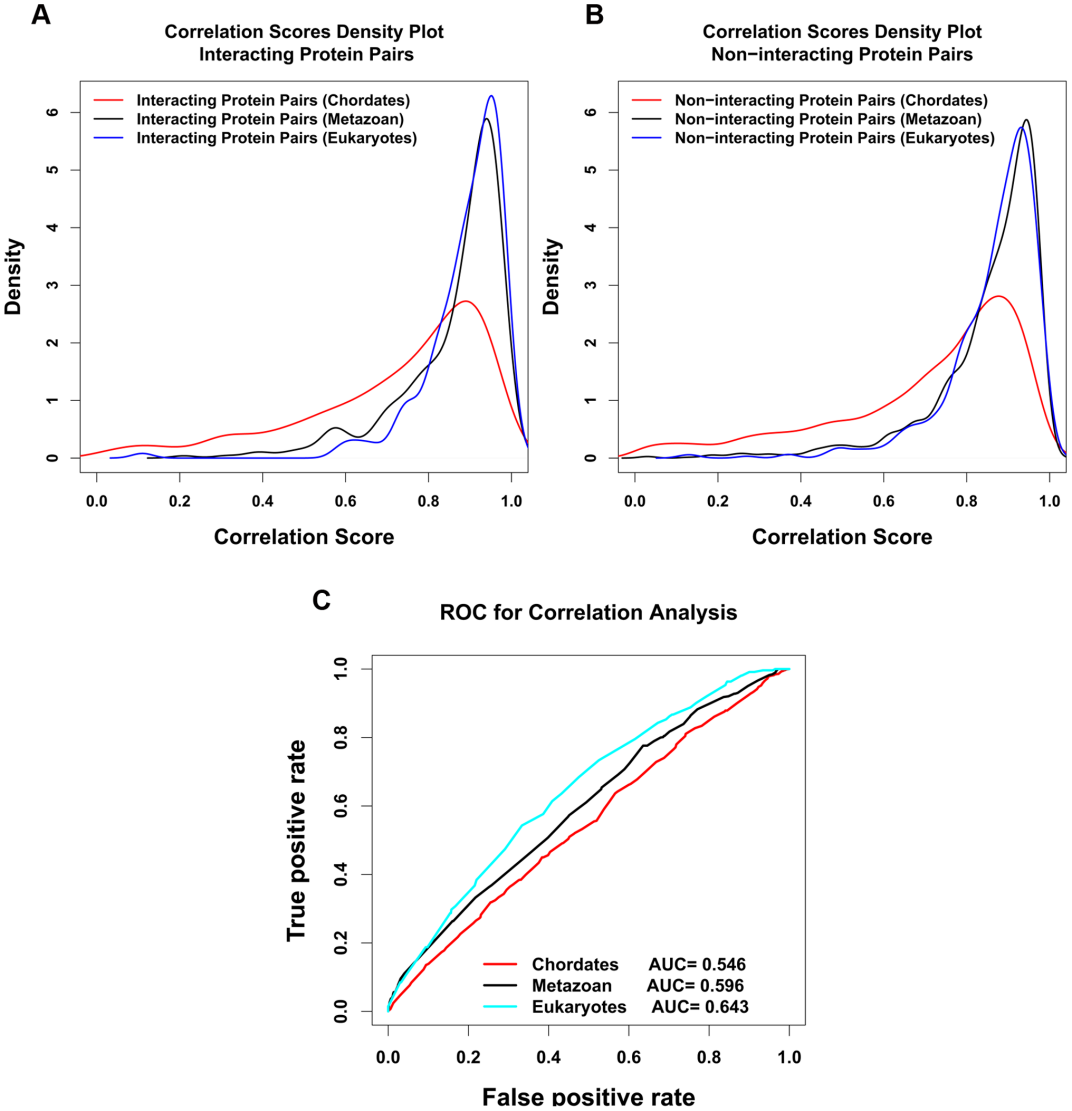
Correlation density plot for interacting (A) and non-interacting (B) Protein pairs of different evolutionary span from Dataset 3. In this plot we separately consider the protein pairs that are conserved only in chordates, the pairs that are conserved across the metazoan but not elsewhere in the eukaryotes, and finally the protein pairs that are distributed across the eukaryotes beyond the metazoan. C). The corresponding ROC plots for the correlation analysis for these 3 different sub-datasets.

We further tested how evolutionary span affects the correlation scores. To do this we divided the results of Dataset 3 according to the evolutionary span of the common species used in the orthology pairs. In Figure 7 we show the results of calculations in which the evolutionary spans were entirely in chordates, entirely in metazoan, or spanned all eukaryotes. This way we have an incrementally increased evolutionary span, and by comparing the correlation scores of interacting protein pairs from these 3 (shown in Figure 7A), we see as the evolutionary span decreases, the peak height of the distribution decreases, while the position of the peak is approximately the same. From the interacting protein pairs from the 3 kingdoms, we also created non-interacting shuffled protein pairs, and the correlation density plot is shown as in Figure 7B. Figure 7C shows ROC plots obtained by comparing interacting and noninteracting pairs in the three subsets of Dataset 3. We see that the AUC score is higher the wider the evolutionary span of the common species of the ortholog pairs. For the widest span, where the ortholog pairs span both metazoan and non-metazoan eukaryotes, the AUC score of 0.643 indicates a fair predictive power, although not as good as Dataset 1.

**Figure 8:**
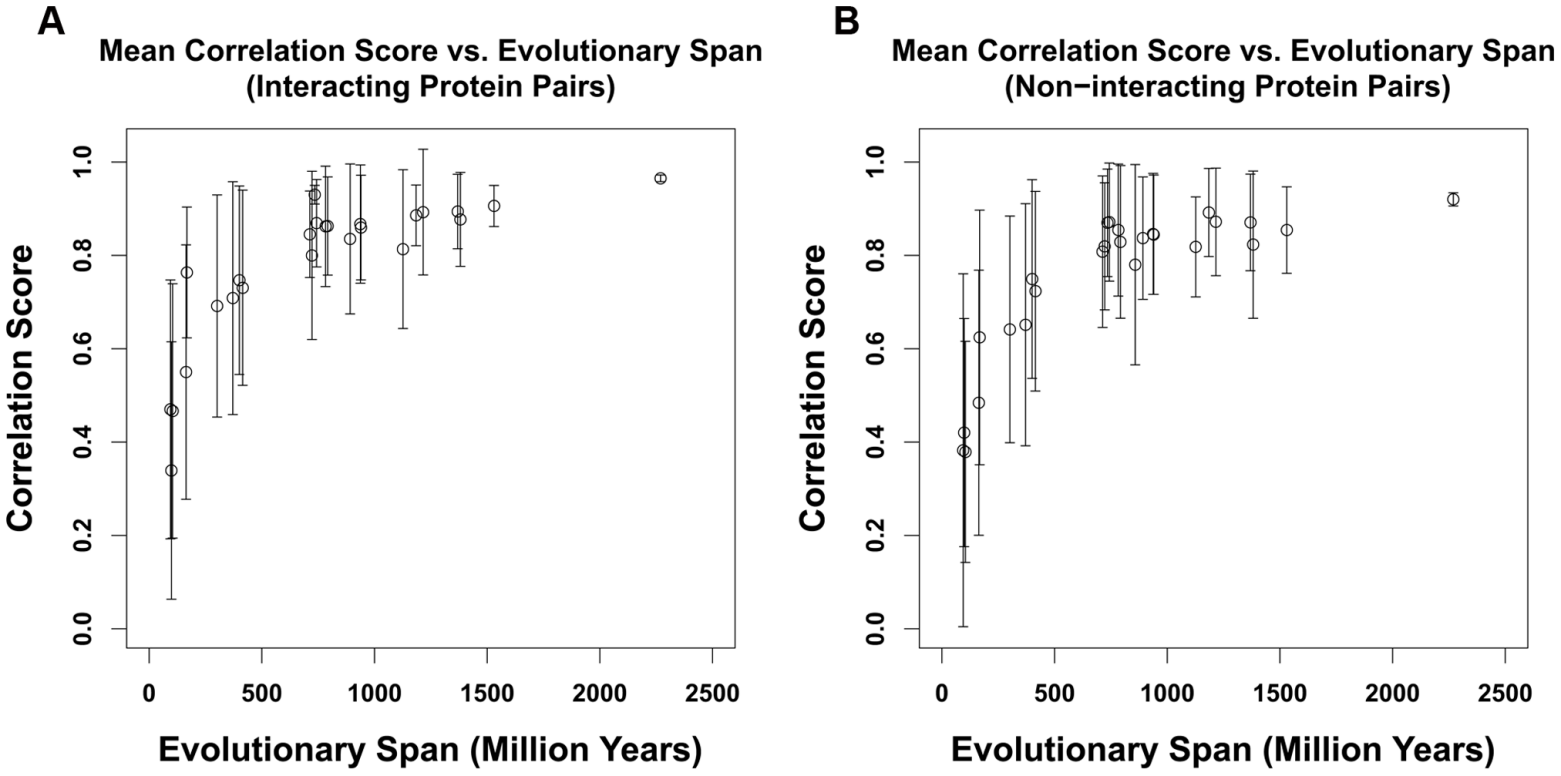
Average correlation vs. evolutionary span for Dataset 3. A). Interacting protein pairs. B). Non-interacting protein pairs. The evolutionary span is defined as the time since last common ancestor for the most distantly related species in the data subset. Correlation scores are mean values for each different evolutionary span, error bar shown as the standard deviation of the correlation scores within respective correlation score range. Range of conservation is defined by the range of the relevant OMA orthology sets. Time since last common ancestor is derived from the TimeTree database [35]. It is seen that the mean score is lower and the standard deviation is larger for data subsets that contain only closely related species.

For another representation of the relationship of correlation score with evolutionary span, we plotted in Figure 8 the correlation score against the time since last common ancestor (as defined in the TimeTree database [35]) for all protein pairs from Dataset 3 in Figure 8. We see that the mean correlation score increases, and the variance decreases, as the time since last common ancestor increases. This is a manifestation of the principle that the statistical significance of similarity patterns in sequences increases with the evolutionary span, perhaps stated most amusingly by Sydney Brenner [36].

### Suggested Points for Using Mirrortree to infer Protein-Protein Interactions in Eukaryotes

- Normalize the evolutionary span among the protein pair orthologous sets to be tested.
- Use as wide an evolutionary span as is available.
- Take into account independent evidence of conservation of interaction, if available.
- If prediction of a binary classification is desired, the peak of the Matthews correlation coefficient is a reasonable default choice for threshold, but the user may shift the threshold up or down depending on whether sensitivity or specificity is more valued.

## Summary and Conclusions

This study was aimed at assessing the mirrortree method for inference of protein-protein interaction, with the goal of understanding how to use it to achieve the most reliable predictions. The major results of our studies are:

1. Over a wide range of degrees of conservation, correlation scores are independent of degree of conservation. However we see lower correlation scores for ortholog pairs that have very high or very low degree of conservation (see Figure 6).
2. Overall correlation scores are higher when wider evolutionary spans are used in the analysis, as shown in Figures 7 and 8. Therefore when comparing protein pairs with each other to infer which is more likely to be interacting, the analysis should be done with orthologs to both pairs covering the same evolutionary span.
3. The method will be more reliable when the particular proteins have a wider evolutionary span, because the signal to noise ratio will be more favorable, as demonstrated in Figure 8. This is a specific example of the general principle that statistical significance of similarity patterns in sequences increases with the evolutionary span covered.
4. For Dataset 1 we find a standard AUC score of the method to be over 0.7, higher than estimated by Hakes et al [21], and much higher than for either Dataset 2 or Dataset 3. The difference between Dataset 1 and Dataset 2 is independent evidence for conservation of interaction across the entire eukaryotic evolutionary span, from human to yeast. In Dataset 3, there is evidence for conservation of interaction between human and mouse, but the evolutionary span of the ortholog sets is not normalized, and the efficacy of the method is thereby compromised.
